# Spatial distribution and characterization of non-apical progenitors in the zebrafish embryo central nervous system

**DOI:** 10.1098/rsob.160312

**Published:** 2017-02-01

**Authors:** Rebecca McIntosh, Joseph Norris, Jon D. Clarke, Paula Alexandre

**Affiliations:** 1 Developmental Biology and Cancer Department, UCL Institute of Child Health, London WC1N 1EH, UK; 2 Division of Surgery and Interventional Science, University College London, London, UK; 3 Department of Developmental Neurobiology, King's College London, London SE1 1UL, UK

**Keywords:** zebrafish, neurogenesis, basal progenitors, Vsx1, live imaging, intermediate progenitors

## Abstract

Studies of non-apical progenitors (NAPs) have been largely limited to the developing mammalian cortex. They are postulated to generate the increase in neuron numbers that underlie mammalian brain expansion. Recently, NAPs have also been reported in the retina and central nervous system of non-mammalian species; in the latter, however, they remain poorly characterized. Here, we characterize NAP location along the zebrafish central nervous system during embryonic development, and determine their cellular and molecular characteristics and renewal capacity. We identified a small population of NAPs in the spinal cord, hindbrain and telencephalon of zebrafish embryos. Live-imaging analysis revealed at least two types of mitotic behaviour in the telencephalon: one NAP subtype retains the apical attachment during division, while another divides in a subapical position disconnected from the apical surface. All NAPs observed in spinal cord lost apical contact prior to mitoses. These NAPs express HuC and produce two neurons from a single division. Manipulation of Notch activity reveals that neurons and NAPs in the spinal cord use similar regulatory mechanisms. This work suggests that the majority of spinal NAPs in zebrafish share characteristics with basal progenitors in mammalian brains.

## 1. Introduction

During vertebrate central nervous system (CNS) development, the majority of neural progenitors divide at the apical surface of the neuroepithelia to self-renew while producing neurons. These apical neural progenitors are initially called neuroepithelial cells and later, due to changes in the expression of markers and in neurogenic potential, become radial glia. Differentiating neurons migrate from the apical ventricular zone and integrate into the mantle zone close to the basal surface of the neuroepithelium, gradually increasing the size of this neuronal layer.

In the mammalian cortex, several other types of progenitors have been reported to divide in non-apical locations. These have been subdivided by their morphology, location of mitoses, renewal capacity and molecular markers. Basal progenitors (also called basal intermediate progenitors) express Tbr2, and lack apical and basal processes [1–5]. In lissencephalic species, the majority of basal progenitors undergo terminal divisions while a small percentage of these progenitors can undergo several rounds of division before producing two neurons [3,6,7]. In gyrencephalic species, the majority of basal progenitors sustain Pax6 expression [8,9] and can generate other non-apical progenitors (NAPs) as well as basal radial glia (bRG) cells. The bRG is a basal progenitor that expresses markers such as Pax6 and Sox2, lacks apical attachment, and is capable of self-renewing and producing basal progenitors and neurons—a property similar to apical neural progenitors [6,8–11]. In the murine cortex, bRG cells quickly undergo self-consuming divisions [12,13]. Others describe subapical progenitors that also divide in basal locations but maintain an apical contact during mitoses [7,11,14]. These subapical progenitors are mainly characterized by undergoing multiple and fast rounds of division.

The non-apical progenitor populations have been widely studied in the mammalian brain, and there is some evidence that their number and subtype could explain differences in brain size and morphology that is observed between species. In mammals, there is a correlation between the number and progenitors subtypes and the number of neurons and size of the brain cortex. An increase in the number of basal progenitors in mouse cortex has been shown to increase brain size [15,16] but does not generate the folding in the cortex. Instead, there is recent evidence that bRGs are the main players in promoting brain growth and cortical folding: bRGs are present in higher numbers in brains of species with gyrified cortex [9–11], and can promote local overgrowth and cortical gyrification by self-renewal of its own population [7,17–19]. In other studies, NAPs have been found in different regions of the developing nervous system of several non-mammalian species. Studies have described the presence of NAPs in the chick retina [20] as well as the thalamus of chick, frog and turtle embryos [21–23]. In zebrafish embryos, NAP populations have been found in the retina [24–26] and also in a region of the CNS with more restricted growth—the spinal cord [27]. This work on zebrafish spinal cord revealed that Vsx1-expressing NAPs divide asymmetrically to generate two distinct neurons, therefore suggesting that NAPs in some systems are not involved in expanding neuronal populations, but rather might contribute to a fast, balanced increase in neuronal diversity from a single progenitor population. However, before we can fully understand the role of NAPs in the relatively small and simple nervous system of the zebrafish we need to quantify their distribution and diversity. Specifically, we need to know whether Vsx1 NAPs [27] are the only population of NAPs in the spinal cord and to quantify the relative proportions of apical and NAPs. Are NAPs randomly distributed throughout the spinal neuraxis or confined to specific locations? In order to investigate the role of NAPs in zebrafish CNS, this work aims to characterize NAP numbers, distribution and diversity in different regions of the CNS, and whether NAPs shared common characteristics with their mammalian counterparts.

Our study reveals that in the zebrafish spinal cord NAPs represent a very small population of the total neural progenitors but that they are very specific in identity and location suggesting a very specific role in development. In the hindbrain, NAPs represent a slightly higher proportion of neural progenitors and are found in three distinct regions of the rhombencephalic neuroepithelium, suggesting a greater diversity than in spinal regions. Like the spinal cord, the embryonic zebrafish telencephalon contains only a very small population of NAPs, but their identity in the telencephalon may be diverse. Vsx1-expressing NAPs represent the large majority of NAPs in the spinal cord and hindbrain. As spinal NAPs expressing Vsx1 have been previously shown to generate one excitatory (V2a) and one inhibitory interneuron (V2b) that integrate into the sensory-locomotor circuit, our observations suggest that Vsx1 NAPs in hindbrain and spinal cord could be important to quickly generate a functional neuronal circuit that regulates zebrafish movements from early stages of embryonic development. We also showed that the majority of NAPs co-express the neuronal marker HuC/D, and in spinal cord, inhibition of the Notch signalling pathway causes a significant increase in numbers of NAPs expressing Vsx1. This suggests that, like basal progenitors in mammalian cortex, the majority of zebrafish NAPs share molecular characteristics and regulatory mechanisms with neurons.

## 2. Material and methods

### 2.1. Animals

Zebrafish wild-type, Tg(Vsx1:GFP) [27], Tg(Olig2:eGFP) [28], Tg(HuC:GFP) [29] and Tg (Tbr2a:dsRed) [30] embryos were raised at 28.5°C in fish water. After 20–24 h post-fertilization (hpf) embryos were maintained in fish water or E2 medium containing 0.003% 1-phenyl-3-(2-thiazolyl)-2-thiourea (PTU; Sigma) to prevent pigment formation.

### 2.2. Immunohistochemistry

We fixed the embryos at 24 hpf, 36 hpf, 48 hpf and 72 hpf in 4%PFA for 2 h at room temperature. For whole-mount immunohistochemistry, we enhanced tissue permeabilization by cryogenic treatments in 24 and 36 hpf embryos and PK treatment in 48 and 72 hpf [31]. For cryogenic treatment, embryos were imbedded in cryogenic buffer (8% sucrose, 5% goat serum, 0.2% gelatin, 1% triton in PBS) at RT for an hour and then incubated twice at −20°C for 3–5 min, until the solution starts forming ice crystals. Embryos were then washed in 0.1% PBT and processed for standard immunohistochemistry.

To label apical and non-apical divisions, we used anti-phospho-histone 3 antibody (rabbit, Upstate Biotech, diluted 1 : 500). To visualize GFP expression in GFP reporter lines, we used anti-GFP antibody (chicken, Abcam, diluted 1 : 1000). The anti-HuC/D antibody (mouse, Invitrogen, 1 : 100) was used as neuronal marker. Nuclei were counterstained with Hoechst (Sigma), Sytox green or Sytox red (Life Technologies).

### 2.3. mRNA injections

Plasmids containing the cDNA coding for CAAX-GFP (membrane marker), H2B-RFP (nuclei marker) and the dominant negative form of Suppressor of Hairless (DN-Su(H)) [32] have been linearized and mRNAs have been synthesized using the mMessage Machine SP6 transcription kit from Ambion. mRNAs were injected either at the one-cell stage for ubiquitous expression or into one blastomere between the 16- to 64-cell stages for mosaic labelling of neural progenitor cells. The mRNA was injected at 10–150 pg per embryo and did not exceed half the volume of a cell.

### 2.4. Zebrafish imaging

The mRNA injected embryos and zebrafish transgene were mounted and imaged as previously described by Alexandre *et al.* [33]. For live-imaging embryos were anaesthetized in MS-222 (Sigma) and kept at 28.5°C. We used SP5 (Leica), LSM 880 (Zeiss), LSM 710 (Zeiss) laser scanning or spinning disk (PerkinElmer) confocal microscopes. For image data analysis, we used ImageJ (http://rsbweb.nih.gov/ij/), Volocity (PerkinElmer) or IMARIS (Bitplane) software.

### 2.5. Statistical analysis

All statistical analyses were performed using Prism7 (GraphPad software). To compare the differences in number of apical and non-apical divisions we used Kruskal–Wallis with Dunn's multiple comparison test. We used *χ*^2^ test to compare the relative proportions of DLB, VMB and SA NAPs in different stages of embryonic development, the relative proportions of neuronal and non-neuronal cell populations, and non-apical and apical divisions in DN-Su(H) and control injected cells.

## 3. Results

### 3.1. Non-apical progenitors are present in specific locations in the zebrafish telencephalon, hindbrain and spinal cord

**Figure 1.**
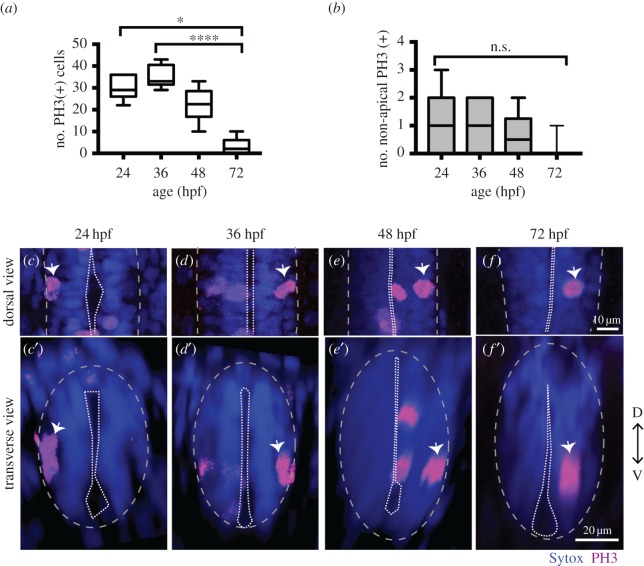
NAPs are present in the zebrafish spinal cord. (*a*) Quantification of all cells in mitosis in a five-somite length of a zebrafish spinal cord at 24, 36, 48, 72 hpf. Data shown as a box-and-whisker plot with the line inside the box representing the mean and whiskers representing minimum and maximum values. The sample size is indicated in the text. Data analysed using Kruskal–Wallis (***p = 0.0001) with Dunn's multiple comparison test (****p < 0.0001, *p = 0.01). (*b*) Quantification of mitoses in non-apical locations in a five-somite length of a zebrafish spinal cord at 24, 36, 48, 72 hpf. Data shown as a box-and-whisker plot with the line inside the box representing the mean and whiskers representing minimum and maximum values. The sample size is indicated in the text. The number of non-apical mitoses does not vary significantly through embryonic development (Kruskal–Wallis, *p* > 0.05) and are rare at 72 hpf. (*c*–*f*) Neural progenitor mitoses revealed by PH3 staining (magenta, indicated by white arrows) in non-apical locations in zebrafish spinal cord at (*c*,*c*′) 24 hpf, (*d*,*d*′) 36 hpf, (*e*,*e*′) 48 hpf and (*f*,*f*′) 72 hpf. Tissue is counterstained with nuclei marker (Sytox, in blue). (*c*–*f*) Single z-slices of dorsal views and (*c*′–*f*′) transverse reconstructions. The grey and white dashed lines outline the basal and apical surfaces of the neuroepithelia, respectively.

**Figure 2.**
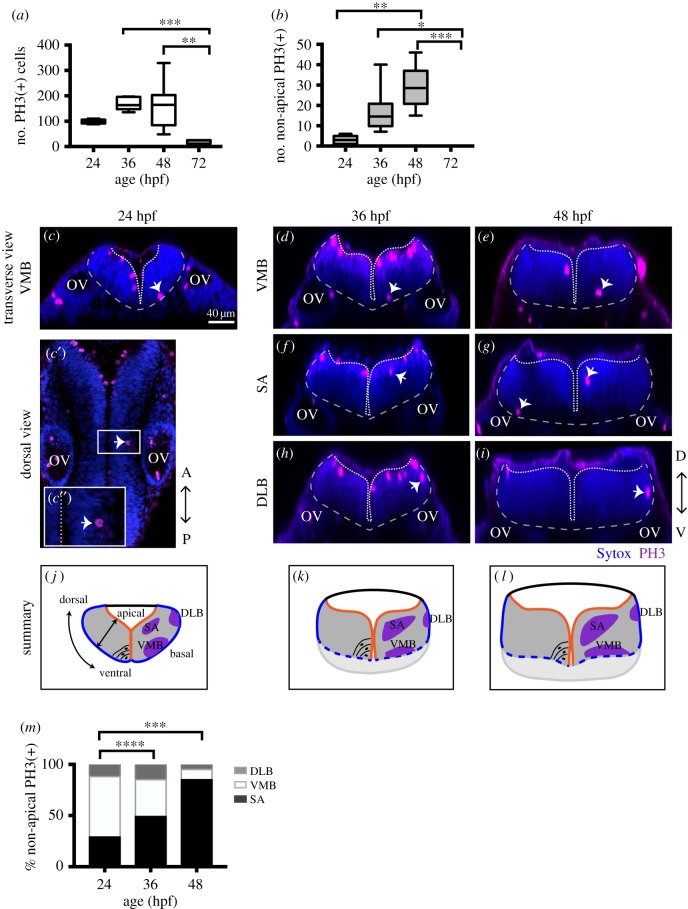
NAPs are restricted to specific regions in the developing hindbrain. (*a*) Quantification of all cells in mitosis in the zebrafish hindbrain at 24, 36, 48, 72 hpf. Data shown as a box-and-whisker plot with the line inside the box representing the mean and whiskers representing minimum and maximum values. The sample size is indicated in the text. Total number of cells in mitosis in the hindbrain (in apical and non-apical locations) decreases significantly at 72 hpf. Data analysed using Kruskal–Wallis (***p = 0.0006) with Dunn's multiple comparison test (**p = 0.0075, ***p = 0.0007). (*b*) Quantification of mitoses in non-apical locations in the zebrafish hindbrain at 24, 36, 48, 72 hpf. Data shown as a box-and-whisker plot with the line inside the box representing the mean and whiskers representing minimum and maximum values. The sample size is indicated in the text. The proportion of non-apical divisions in the hindbrain significantly increases between 24 and 48 hpf, but they are absent at 72 hpf. Data analysed using Kruskal–Wallis (****p < 0.0001) with Dunn's multiple comparison test (*p = 0.0124, **p = 0.0038,***p = 0.0002). (*c*–*i*) Neural progenitor mitoses revealed by PH3 staining (magenta, indicated by white arrows) in non-apical locations in zebrafish hindbrain at (*c*–*c*″) 24 hpf, (*d*,*f*,*h*) 36 hpf and (*e*,*g*,*i*) 48 hpf. Tissue is counterstained with nuclei marker (Sytox, in blue). (*c*′,*c*″) Single z-slice of a dorsal view of zebrafish hindbrain at low magnification; in (*c*′) a white arrow indicates an NAP mitosis, in (*c*″) higher magnification of white box in (*c*′). (*c*,*d*–*i*) Transverse optical sections of the zebrafish hindbrain show the position of NAP mitoses along the dorsoventral and mediolateral axis at (*c*–*c*″) 24 hpf, (*d*,*f*,*h*) 36 hpf and (*e*,*g*,*i*) 48 hpf. (*c*–*i*) At 24, 36 and 48 hpf NAP mitoses can occur in (*c*,*d*,*e*) ventral medial basal (VMB), (*f*,*g*) subapical (SA) and (*h*,*i*) dorsolateral basal (DLB) regions of the hindbrain neuroepithelium. Observations are summarized in (*j*–*l*). (*m*) The relative proportions of VMB, SA, DLB NAPs populations in the hindbrain at 24, 36 and 48 hpf are shown in the stacked bar diagram. Data analysed using a *χ*^2^ test (****p = 0.0001, ***p = 0.0006). In (*c*,*c*″,*d*–*i*) the basal and apical surfaces of the neuroepithelia are outlined by grey and white dashed lines respectively. OV, otic vesicle; 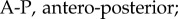 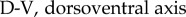.

**Figure 3.**
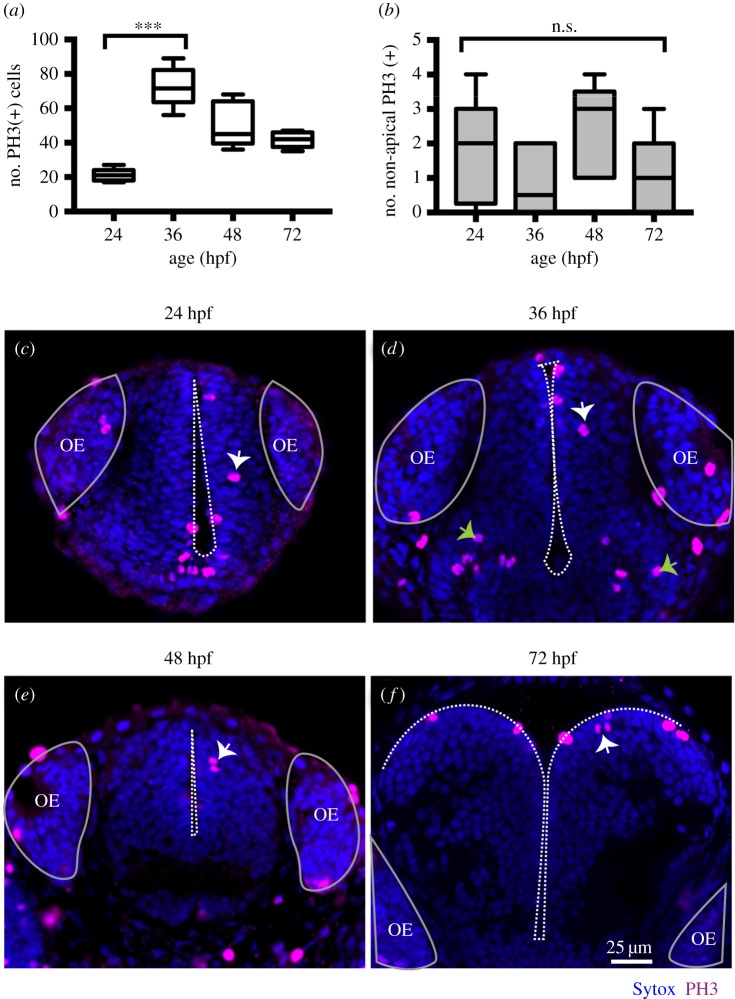
(*a*) Quantification of all cells in mitosis in the zebrafish telencephalon at 24, 36, 48, 72 hpf. Data shown as a box-and-whisker plot with the line inside the box representing the mean, and whiskers representing minimum and maximum values. The sample size is indicated in the text. The number of cells in mitosis increases significantly at 36 hpf. Data analysed using Kruskal–Wallis (***p = 0.0004) with Dunn's multiple comparison test (*p* = 0.0002). (*b*) Quantification of all cells in mitosis in the zebrafish telencephalon at 24, 36, 48, 72 hpf. Data shown as a box-and-whisker plot with the line inside the box representing the mean and whiskers representing minimum and maximum values. The sample size is indicated in the text. The number of NAP mitoses does not vary significantly from 24 to 72 hpf. Data analysed using Kruskal–Wallis (*p* = 0.18). (*c*–*f*) Neural progenitor mitoses revealed by PH3 staining (magenta, indicated by white arrows) in non-apical locations of zebrafish telencephalon at (*c*) 24 hpf, (*d*) 36 hpf, (*e*) 48 hpf, (*f*) 72 hpf. Tissue is counterstained with nuclei marker (Sytox, in blue). Images are single z-slices of a transverse view of zebrafish telencephalon at (*c*) 24 hpf, (*d*) 36 hpf, (*e*) 48 hpf, (*f*) 72 hpf. Green arrows in (*d*) indicate clusters of mitoses in hypothalamic territory that were not analysed. A white dash line outlines apical surface or ventricle of zebrafish telencephalon. OE, olfactory epithelium.

In order to detect and characterize NAP populations in the spinal cord, hindbrain and telencephalon of the developing zebrafish embryo, we immuno-labelled cells in mitosis with an antibody against phospho histone 3 (PH3). To outline the shape of the neural tissue, we counterstained cell nuclei with Sytox. Analysis of position and quantification of neural progenitors in division was carried out at several stages of embryonic development from 24 hpf to 72 hpf. High-resolution z-stacks were captured of the neural tube from a dorsal view, allowing us to visualize the whole tissue and to reconstruct data in transverse section. From these data, we observed that some neural progenitors divide in non-apical locations in all three regions of the CNS analysed (figures 1–3). We define non-apical divisions if they occur at least one cell nuclei distance from the apical surface. These non-apical divisions are a small percentage of the total number of dividing neural progenitors with the large majority dividing at the apical surface.

In spinal cord we observed the non-apical divisions represent around 3% of all divisions at 24 to 48 hpf and are very rare by 72 hpf (we have found a single NAP division at 72 hpf, *n* = 16 embryos) (figure 1*a*,*b*). We observed on average one non-apical division per five-somite length of spinal cord from 24 to 48 hpf (mean ± s.e.m.: 24 hpf, 1 ± 0.43, 3.4% of total number of divisions, *n* = 7 embryos; 36 hpf, 1.11 ± 0.31, 3.2% of total number of divisions, *n* = 9 embryos; 48 hpf, 0.67 ± 0.33, 3.3% of total number of divisions, *n* = 7 embryos) (figure 1*b*–*f*′). Reconstructions in the transverse plane show that non-apical divisions are located within the ventrolateral quadrant of the spinal cord throughout this period of embryonic development (figure 1*c*′–*f*′).

NAPs are also found in the hindbrain, and there is a significant increase in their number from 24 hpf to 48 hpf (mean ± s.e.m. 24 hpf: 3 ± 0.82, 3% total number of divisions, *n* = 7 embryos; 36 hpf: 11.75 ± 3.9, 7% of total number of divisions, *n* = 7 embryos; 48 hpf: 28.9 ± 3.6, 18% of the total number of divisions, *n* = 7 embryos) (figure 2*a*,*b*). At 72 hpf the total number of divisions is significantly reduced and we were unable to find non-apical divisions in the hindbrain (*n* = 5) (figure 2*a*,*b*). Transverse reconstructions of the hindbrain show that NAP divisions are spatially restricted in this region (figure 2*c*–*l*). One population is present at the basal extremity of the mantle zone in the ventromedial quadrant of the tissue (ventromedial basal, VMB) (figure 2*c*–*e*,*j*–*l*). A second population of NAPs is found at subapical (SA) locations (figure 2*f*,*g*,*j*–*l*); and a third NAP population positions the mitotic nuclei at the basal surface of the hindbrain in the dorsolateral quadrant (dorsolateral basal, DLB) (figure 2*h*,*i*,*j*–*l*). The relative proportions of DLB, VMB and SA NAP populations vary significantly between 24 hpf and later stages of embryonic development (figure 2*m*). During embryonic development, the SA NAP population expands while the VMB decreases, suggesting that surface NAPs are favoured through time.

In the zebrafish, telencephalon non-apical mitoses were detected at 24 hpf (mean ± s.e.m.: 24 hpf: 1.9 ± 0.18, 6.9% of total number of divisions, *n* = 8 embryos) (figure 3*a*,*b*) and their number does not significantly alter during embryonic development (36 hpf: 0.8 ± 0.4, *n* = 6, 1.2% of total number of divisions; 48 hpf: 2.4 ± 0.6, 4.8% of total number of divisions, *n* = 5 embryos, 72 hpf: 1 ± 0.6, 2.4% of total number of divisions, *n* = 5 embryos) (figure 3*b*). In contrast with other brain regions, we find the majority of non-apical divisions reside in subapical locations in the telencephalon (figure 3*c*–*f*).

In summary, these observations reveal the different spatial distribution of NAP mitoses in the zebrafish spinal cord, hindbrain and telencephalon up to 72 hpf, suggesting that different NAPs populations maybe present in the developing zebrafish CNS.

### 3.2. Molecularly distinct populations of non-apical progenitors are present in the zebrafish central nervous system

To determine the identity of NAP populations in the zebrafish CNS, we have analysed NAP mitoses in transgenic embryos that report the expression of Vsx1, Olig2 and Tbr2 transcription factors, which have been previously associated with NAPs in zebrafish or mammalian systems. In the zebrafish spinal cord, Vsx1 is exclusively expressed by a specific population of NAPs that generates two distinct interneurons, V2a and V2b, at each division [27]. Olig2 is known to label motor neurons, motor neuron progenitors and non-apically dividing oligodendrocyte precursor cells (OPCs) in zebrafish [34,35]. Tbr2 is a T-box transcription factor and well-known marker for mammalian basal progenitors [5], which typically undergo self-consuming divisions generating two neurons. However, in non-mammalian organisms, Tbr2 expression has been reported in telencephalic neurons in chick and in zebrafish [23,36], and only a small proportion of Tbr2+ cells (less than 0.2%) seem to co-express the mitotic marker PH3 [23].

**Figure 4.**
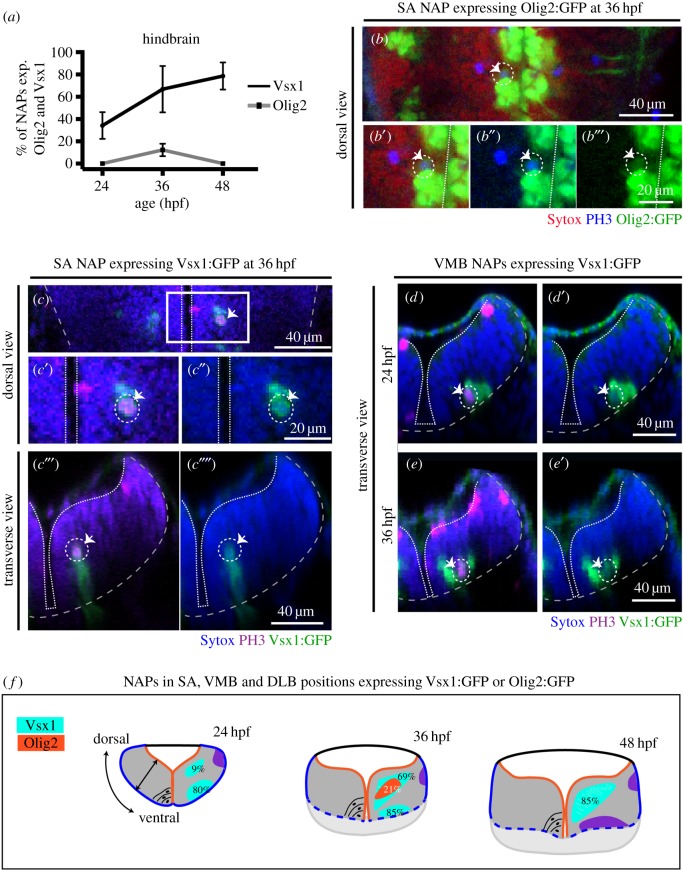
Vsx1 and Olig2 are expressed by distinct subpopulations of NAPs in zebrafish developing hindbrain. (*a*) Quantification of non-apical mitoses expressing Vsx1:GFP and Olig2:GFP in hindbrain at 24, 36, 48 hpf. (*b–b*″) Single z-slice of a dorsal view of hindbrain at 36 hpf showing NAP in mitosis in SA position expressing Olig2:GFP (indicated by a dashed circle and white arrow). (*c*–*c*′′′′) Single z-slice of a dorsal (*c*–*c*″) and transverse (*c*′″,*c*′′′′) views of hindbrain at 36 hpf, showing NAP in mitosis in SA position expressing Vsx1:GFP (indicated by a dashed circle and white arrow). (*d*–*e*′) Transverse hindbrain sections at 24 and 36 hpf showing NAPs in mitosis in VMB positions expressing Vsx1:GFP (indicated by a dashed circle and white arrow). (*f*) Quantification of NAPs in SA, VMB and DLB positions in the zebrafish hindbrain, expressing Olig2:GFP and Vsx1:GFP at 24, 36 and 48 hpf. Magenta (blue in (*b*–*b*′″)) is PH3 staining and blue (red in (*b*–*b*′″)) is Sytox nuclear counter stain. The grey and white dashed lines outline the basal and apical surfaces of the neuroepithelia, respectively.

In the hindbrain, we observed that NAPs can express Olig2 and Vsx1 but not Tbr2. Olig2 is only expressed by a small proportion of NAPs at 36 hpf (NAPs expressing Olig2 at 24 hpf: 0/33 (0%), *n* = 4 embryos, 36 hpf: 11/94 (12%), *n* = 5 embryos, 48 hpf: 0/49 (0%), *n* = 5 embryos) (figure 4*a*–*b*″′), while Vsx1 is expressed by NAPs during the first 2 days of embryonic development (NAPs expressing Vsx1 at 24 hpf: 9/26 (34%), *n* = 4 embryos, 36 hpf: 51/78 (67%), *n* = 4 embryos, 48 hpf: 35/44, (79%), *n* = 5 embryos) (figure 4*a*,*c*–*e*′). The analysis of Vsx1 and Olig2 expression by the distinct populations of NAPs (SA, VMB, DLB) revealed that Vsx1 can be expressed by NAPs in SA positions from 24 to 48 hpf (SA NAPs expressing Vsx1 at 24 hpf: 1/11 (9%), *n* = 4 embryos, 36 hpf: 22/32 (69%), *n* = 4 embryos, 48 hpf: 33/39 (85%), *n* = 5 embryos) (figure 4*c*–*c*′′′′,*f*) and by a large majority of NAPs in the VMB positions from 24 to 36 hpf (VMB NAPs expressing Vsx1 at 24 hpf: 8/10, 80%, *n* = 4 embryos, 36 hpf: 29/34 (85%), *n* = 4 embryos, 48 hpf: 0/3 (0%), *n* = 5 embryos) (figure 4*d*–*f*). Olig2, however, is only found expressed at 36 hpf in NAPs confined to SA positions (figure 4*b*-*b*′′′,*f*). These data reveal that different populations of NAPs in the hindbrain express molecularly distinct markers and are therefore likely to produce different cellular outputs. However, it remains unknown whether, for example, NAPs expressing Vsx1 in SA or VMB positions produce similar progeny.

**Figure 5.**
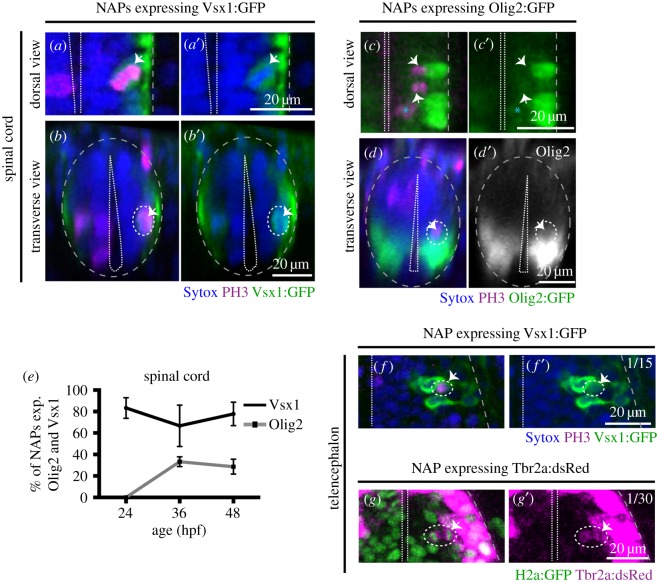
Vsx1, Olig2 and Tbr2 are expressed by subpopulations of NAPs in zebrafish spinal cord and telencephalon. (*a*,*a*′) Horizontal and (*b*,*b*′) transverse sections of spinal NAP in mitosis (white arrow) expressing Vsx1:GFP at 36 hpf. (*c*,*c*′) Horizontal and (*d*,*d*′) transverse section of spinal cord with a NAPs in mitosis (white arrow) expressing low levels of Olig2:GFP at 36 hpf. (*e*) Quantification of non-apical mitoses expressing Vsx1:GFP (black) or Olig2:GFP (grey) in spinal cord at 24, 36, 48 hpf. (*f*,*f*′) Single z-slice of NAP in mitosis (white arrow) expressing Vsx1:GFP in the telencephalon at 24 hpf. (*g*,*g*′) Single z-slice of NAP in mitosis (white arrow) expressing Tbr2a:dsRed in the telencephalon at 24 hpf. Green labels cell nuclei and magenta labels Tbr2 transgene. In (*a*–*f*′), magenta is PH3 staining and blue is Sytox nuclear counter stain. The grey and white dashed lines outline the basal and apical surfaces of the neuroepithelia, respectively.

In the spinal cord at 24 hpf, we observed that almost all non-apical mitoses expressed Vsx1:GFP (5/6, 83.3% of NAPs express Vsx1:GFP, *n* = 5 embryos) (figure 5*e*). At 36 and 48 hpf the majority of NAPs expressed Vsx1:GFP (36 hpf: 4/6, 67% of NAPs express Vsx1:GFP, *n* = 4 embryos; 48 hpf: 7/9, 78% of NAPs express Vsx1:GFP, *n* = 5 embryos) (figure 5*a*–*b*′,*e*) and a small population of NAPs co-express Olig2:GFP (36 hpf: 3/9, 33% of NAPs express olig2:GFP; *n* = 5 embryos; 48 hpf: 2/7, 28.5% of NAPs express Olig2:GFP; *n* = 6 embryos) (figure 5*c*–*d*′,*e*; electronic supplementary material, movie S1). From these data we can conclude that in the spinal cord the majority of NAPs at 24 to 48 hpf express Vsx1:GFP (figure 5*a*–*b*′,*e*), while a small population of NAPs expressing Olig2:GFP only emerges at 36 hpf and 48 hpf (figure 5*c*–*e*). The appearance of NAPs expressing Olig2 at 36 hpf coincides with the timing by which the first OPCs emerge in the spinal cord [34,35].

In the telencephalon, the large majority of non-apical divisions express neither Vsx1, Tbr2 nor Olig2. We observed a single subapical division expressing Vsx1 at 24 hpf (1/15, 6.6% of all non-apical divisions, *n* = 8 embryos per embryonic stage) (figure 5*f*,*f*′) but none at later stages of embryonic development (36 hpf: 0/13 (0%), *n* = 5 embryos, 48 hpf: 0/11 (0%), *n* = 5 embryos, 72 hpf: 0/7 (0%), *n* = 5 embryos). We were also unable to find Tbr2:DsRed expressing NAPs in the telencephalon of fixed embryos (NAPs expressing Tbr2 at 24 hpf: 0/5 (0%), *n* = 4 embryos, 36 hpf: 0/11 (0%), *n* = 4 embryos, 48 hpf: 0/5 (0%), *n* = 4 embryos, 72 hpf: 0/3 (0%), *n* = 3 embryos). To improve our chances of finding Tbr2 expressing NAPs, we changed our experimental approach and used zebrafish live imaging to monitor NAPs divisions in the Tbr2:DsRed transgenic embryos. To visualize mitoses, embryos have been injected with mRNA encoding nuclear-GFP. The imaging started at 22 hpf and continued for several hours using a confocal microscope. Using this approach, we were able to detect a single division expressing Tbr2 (1/30 NAP divisions, 3.3% of all non-apical divisions, *n* = 4 embryos) (figure 5*g*,*g*′), suggesting that Vsx1- and Tbr2-expressing cells are a very small proportion of NAPs in the telencephalon, thus the molecular signature of most zebrafish telencephalic NAPs remains to be determined.

These results confirm the presence of several molecularly and spatially distinct NAPs in zebrafish spinal cord, hindbrain and telencephalon. These regional and molecularly distinct NAPs are likely to generate different neuronal subtypes. For example, Vsx1-expressing NAPs can potentially generate distinct interneurons while Olig2 positive NAPs could generate OPCs [34,35], motor neurons [34] or interneurons if similar to Olig expressing precursors in the mammalian telencephalon [37]. Vsx1-expressing cells were the predominant NAP population in the zebrafish hindbrain and spinal cord, although in the hindbrain and telencephalon there is a large subpopulation of NAPs that remains molecularly unidentified.

### 3.3. Non-apical progenitors generate two neurons

**Figure 6.**
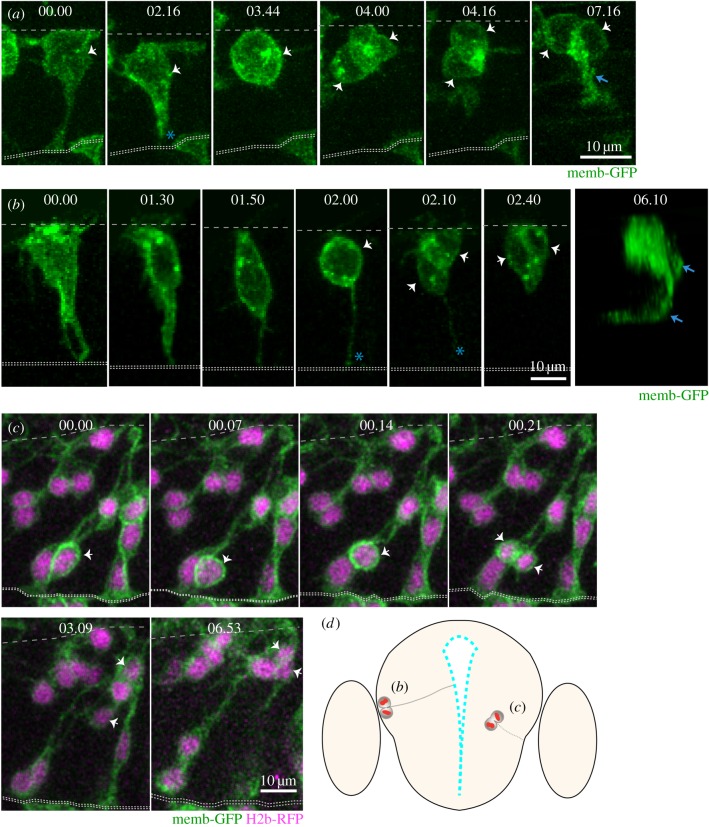
Live imaging reveals distinct behaviours of NAPs in zebrafish CNS (time points shown in hours and minutes). (*a*) Images from confocal time-lapse showing NAP dividing at the basal surface of the neuroepithelium in the spinal cord and generating two daughter neurons at 24 hpf. The NAP loses apical contact prior to division. White arrows in time points 03.44 and 04.00 indicate the dividing NAP and then its two daughter cells. The basal and apical surfaces of the neural tube are outlined by dashed lines at the top and bottom of this time sequence, respectively. An axon is evident (blue arrow) by time point 07.16. (*b*) Images from confocal time-lapse showing NAP dividing at the basal surface of telencephalic neuroepithelium (white arrow in time point 02.00) at 24 hpf and generating two daughter cells (white arrows in time points 02.10 and 02.40) that will become neurons. A three-dimensional reconstruction of the image at time point 06.10 shows that two axons have been formed (blue arrows). This NAP retains the apical attachment during mitosis but releases the apical surface soon after division (blue asterisk in time points 02.00 and 02.10). Basal surface shown by dashed line at top of the image and apical surface shown by two dotted lines at bottom. (*c*) Images from confocal time-lapse showing NAP dividing in a subapical position of telencephalic neuroepithelium at 30 hpf (white arrows in time points 00.14 and 00.21). This division generates two daughter cells that adopt a round shape and move basally into neuronal mantle layer (white arrows in time points 03.09 and 06.53). This NAP does not retain the apical contact during division. A diagrammatic transverse section of the telencephalon is shown in (*d*) to illustrate location of NAP mitoses shown in (*b*) and (*c*). All images are projected images from confocal z-stacks.

The analysis of non-apical mitoses shows that different populations of NAPs are present in the zebrafish CNS. To determine whether these NAPs have different morphologies and self-renewal or neurogenic potential, we followed individual neural progenitors using live imaging in the spinal cord and telencephalon of zebrafish embryos. These regions are better suited for live imaging at these stages of embryonic development, as the hindbrain NAPs are located deeper in the neural tissue and are more difficult to image. We generated mosaically labelled neural cells by injecting a single cell at the 16–32 cell stage of development with mRNA encoding membrane-GFP. We imaged these labelled cells from 22 hpf for 24 h using a confocal microscope. Although NAPs represent only a very small percentage of total progenitors, we were able to find nine NAPs from 17 embryos imaged. In the spinal cord, we monitored seven NAPs dividing at the basal surface of the neuroepithelia (figure 6*a*; electronic supplementary material, movie S2). At the time of mitosis, these cells have no attachment to the apical surface and these NAPs all generated two daughter neurons (image sequence in figure 6*a*). Their neuronal fate can be confirmed by the observation of axon extension (arrows in the last time point of image sequence in figure 6*a*). The axons of neuronal sisters can be difficult to distinguish as they grow often together. To confirm that NAPs progeny in the spinal cord are indeed generating two neurons we imaged the Vsx1:GFP transgene, which represents 83.3% of all NAPs dividing in the spinal cord at this stage of embryonic development. Using this approach we were able to confirm that pairs of neurons resulting from Vsx1:GFP expressing NAPs grow two axons in close vicinity to each other (thus only distinguishable intermittently in electronic supplementary material, movie S3). In the telencephalon, we observed the mitosis of two NAPs (figure 6*b*,*c*; electronic supplementary material, movies S4 and S5). One retained an apical attachment during mitosis (indicated by blue asterisk in figure 6*b*; electronic supplementary material, movie S4) while the other detached from the apical surface before undergoing mitosis in a subapical position (arrows in figure 6*c*; electronic supplementary material, movie S5). Both of these telencephalic NAPs generated two neuronal daughters but their expression profile was not assessed.

These data reveal the cellular morphology of NAPs prior to, during and after cell division in the zebrafish CNS, and indicate that zebrafish NAPs can adopt different cell shapes. Whether these morphological differences are relevant for daughter fate decisions remains unknown in both zebrafish and mammalian systems. The observed zebrafish NAPs are neurogenic and undergo terminal divisions to produce two neurons. So far we have been unable to detect self-renewing NAPs in zebrafish CNS, probably because they are rare, non-existent or are located in regions difficult to image.

### 3.4. Non-apical progenitors express neuronal markers and their generation is regulated by Notch signalling

**Figure 7.**
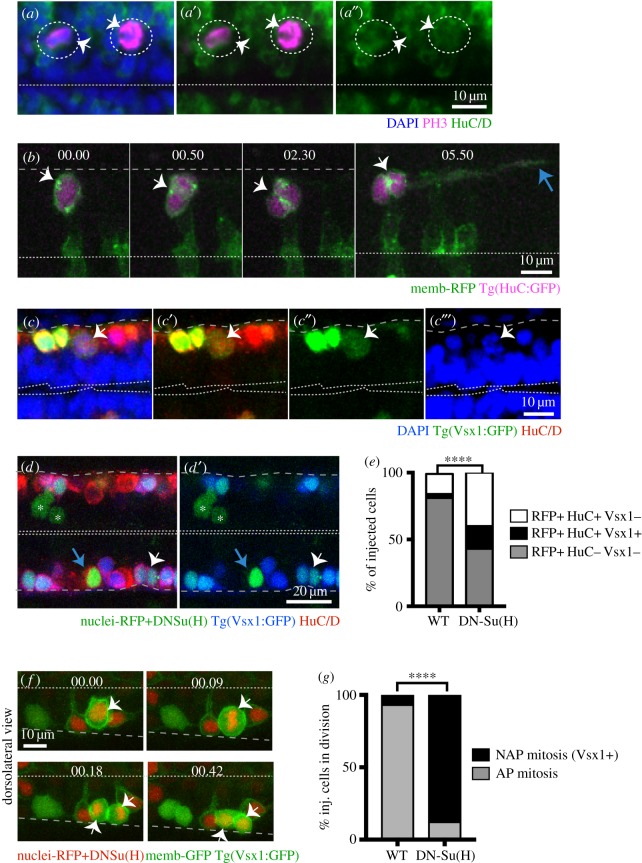
NAPs and neurons share similar molecular mechanisms. (*a*–*a″*) Two NAPs (PH3+, magenta) divide away from the apical surface of the hindbrain and express the neuronal marker HuC/D (green). PH3 and HuC/D have been detected by immunohistochemistry while the overall tissue has been counterstained with nuclei marker (Sytox, in blue). Dotted circles and white arrows indicate the dividing progenitors. Dashed line at the bottom of image indicates apical surface. (*b*) Time-lapse sequence from projected confocal stack shows NAP in spinal cord expressing membrane-RFP (green) and Tg(HuC:GFP) (magenta) while undergoing mitosis (white arrow at time point 00.00 and 00.50) at basal surface of neuroepithelium. Later (at 5h50) the two daughter cells elongate axons (blue arrow) confirming the neuronal fate. Dashed line at the bottom of image indicates apical surface. (*b*,*f*) Time points shown in hours and minutes. (*c*–*c*′″) NAPs in the spinal cord expressing Vsx1 (Vsx1:GFP+, green) express the neuronal marker HuC/D (red) during mitosis as indicated by the white arrows. The dividing cell at top of the image is in prophase. HuC/D has been detected by immunohistochemistry and tissue counterstained with nuclei marker (Sytox, in blue). Single confocal slice from dorsal view of spinal cord. Double dashed line at the bottom of image indicates apical surface. (*d*–*e*) Mosaic overexpression of Dn-Su(H) (green labelled cells) in the spinal cord increases Vsx1 expressing (Vsx1+) NAP population (white arrows in (*d*) and (*d*′)). Cells expressing nuclear-RFP alone (control) or with Dn-Su(H) mRNAs ((*d*,*d*′) in green) can be neuronal NAPs derived (HuC/D+ and Vsx1:GFP, triple labelled in green/blue/red, indicated by white arrow), neuronal non-NAPs derived (HuC/D+ but Vsx1:GFP-, double labelled in green/red, indicated by blue arrow), non-neuronal cells (neuronal progenitors) (Vsx1:GFP- and HuC/D-, labelled in green, indicated by white asterisks). (*e*) The relative proportions of these distinct cell populations in control or Dn-Su(H) expressing embryos are shown in the stack bar diagram and reveal an increase in neuronal cell types (NAPs (RFP+/HuC/D+/Vsx1+) and non-NAPs (RFP+/HuC/D+/Vsx1-) derived) at the expense of non-neuronal cell types (progenitors (RFP+/HuC/D-/Vsx1-)). Data analysed using a *χ*^2^ test (****p < 0.0001). (*f*) Image sequence showing a dividing NAP expressing membrane-GFP, nuclei-RFP, DN-Su(H) and Vsx1:GFP in zebrafish spinal cord (white arrows). Apical surface shown by white dashed line at top of the image and basal surface shown by grey dashed line at bottom. (*g*) The relative proportions of mitoses occurring at the apical (AP mitosis) and non-apical (NAP mitosis/Vsx1+) locations in the spinal cord of control and DN-Su(H) expressing embryos are shown in the stack bar diagram. (*f*–*g*) Cells expressing DNSu(H) (image sequence in (*f*)) preferentially divide in non-apical positions and express Vsx1:GFP. (*g*) Data analysed using a *χ*^2^ test (****p < 0.0001).

Mammalian studies have revealed that basal progenitors that undergo terminal division express neuronal markers [2,18]. In zebrafish, all NAPs that we followed by live imaging produced two neurons, suggesting they may share some other characteristics with mammalian basal progenitors. To test whether this is the case, we immunostained embryos against the pan neuronal marker HuC/D and also used live imaging of the neuronal reporter transgenic line tg(HuC:GFP). Both approaches showed that during division NAPs already express the neuronal marker HuC/D (figure 7*a*,*b*). These data reveal that zebrafish spinal and hindbrain NAP populations express neuronal markers, which suggest that NAPs and neurons may be regulated by very similar molecular mechanisms.

To confirm that NAPs and neurons share some regulatory mechanisms, we tested whether forced neuronal differentiation would concurrently increase the number of neurons and NAPs in zebrafish spinal cord. The expression of DN-Su(H) in neural progenitors has been previously shown to inhibit the activation of the Notch signalling pathway and promote neuronal differentiation [32,38]. We therefore predicted that if NAPs and neurons share similar regulatory mechanisms, the overexpression of DN-Su(H) in zebrafish CNS should also increase the number of NAP divisions and NAP progeny. To prevent disruption of neural tube morphogenesis we performed mosaic injections of mRNAs coding for DN-Su(H) (to promote neuronal differentiation) and nuclear-RFP (to lineage label injected cells) (example in figure 7*d*,*d*′). Control embryos were only injected with mRNA coding for nuclear-RFP. In the spinal cord, the expression of Vsx1:GFP transgene allows the distinction between apical and NAPs, as Vsx1:GFP is expressed by NAPs and NAPs progeny but never by apical progenitors.

To determine whether the proportion of NAPs and apical progenitors derived neurons increase with the expression of DN-Su(H) in the spinal cord, we used embryos expressing Vsx1:GFP transgene and label them with the neuronal marker HuC/D (illustrated in figure 7*d*,*d*′). This combination of molecular markers allows the distinction between NAP-derived neurons (nuclear-RFP+/Vsx1:GFP+/HuC/D+, white arrow in figure 7*d*,*d*′), apical progenitor-derived neurons (nuclear-RFP+/HuC/D+/Vsx1:GFP-, indicated by blue arrow in figure 7*d*,*d*′) and non-neuronal cells (nuclear-RFP+/HuC/D-/Vsx1:GFP-, indicated by asterisks in figure 7*d*,*d*′). First, this analysis revealed that all cells expressing Vsx1:GFP, including the ones in mitoses (NAPs Vsx1+) (white arrow in figure 7*c*–*c*′″), are neurogenic as they all co-express HuC/D. In addition, we quantified the proportion of neuronal fates (HuC/D+ and/or Vsx1:GFP+) and confirmed that their number has significantly increased in DN-Su(H)-injected embryos when compared with control embryos (58.9% (228/387 cells) of DN-Su(H)-expressing cells are HuC/D positive, *n* = 6 embryos while only 20.4% (142/696 cells) of control cells are HuC/D positive; *n* = 6 embryos), although the non-neuronal cell population (nuclear-RFP+/HuC/D-/Vsx1:GFP-) is significantly reduced in DN-Su(H) (41.1%; 159/387 cells, *n* = 6 embryos) when compared with the control situation (79.6%; 554/696 cells, *n* = 6 embryos)(figure 6*e*). This observation suggests that the significant increase in neuronal differentiation observed in embryos expressing DN-Su(H) occurs at the expense of undifferentiated progenitor populations (nuclear-RFP+/HuC/D-/Vsx1:GFP-) (figure 6*e*). To confirm that the expansion of Vsx1:GFP neuronal population following DN-Su(H) expression results from NAPs divisions, we use live imaging to monitor cell divisions in the Vsx1:GFP transgene. These experiments revealed that in contrast with control embryos, cells expressing DN-Su(H) rarely divide at the apical surface (2/17 divisions) of the neuroepithelia, and instead undergo mitoses in non-apical locations while expressing Vsx1:GFP (15/17) (figure 7*f*,*g*; electronic supplementary material, movie S6).

These data demonstrate that NAP populations express neuronal markers and expand their population in response to differentiating signals. It also confirms, at least in zebrafish spinal cord, that NAPs share some common regulatory mechanisms with neurons.

## 4. Discussion

NAPs generate most of the neurons in the mammalian cortex, and their evolution has been proposed as a key feature that has allowed the huge expansion and sophistication of this brain region [7,9]. To understand whether NAPs may also contribute to the generation of less expansive and less sophisticated brain regions, we have analysed NAPs in the embryonic brain and spinal cord of the zebrafish. Despite the relatively small size and simplicity of the teleost CNS, we uncovered NAPs in telencephalon, hindbrain and spinal cord, and our results suggest that multiple types of NAPs are present in this relatively simple system. Furthermore, embryonic zebrafish NAPs constitute only a very small percentage of the total number of neural progenitors in the telencephalon, hindbrain and spinal cord, thus neurogenesis is dominated by apical progenitors in these brain regions. This suggests that embryonic teleost NAPs do not contribute significantly to a large expansion of neuronal numbers and instead that their presence may be related to neuronal diversity. The majority of NAPs that have been monitored by live imaging generated two neurons following division.

In zebrafish spinal cord, although NAPs are a small population, we suggest they play three distinct roles. First, because each NAP division produces two neurons they are expanding the neuronal population maximally in a single mitotic event. Second, they create neuronal diversity by generating asymmetrically fated neuronal daughters [27] (in this case one V2a and one V2b interneuron). Third, because the V2a neuron is excitatory while V2b is inhibitory, they produce a balanced output of excitatory and inhibitory neurons that may be critical in locomotor circuit formation. Our data suggest Vsx1-expressing NAPs are the largest population of neuron-producing NAPs in the zebrafish spinal cord, further indicating that they play a distinct role in circuit production. However, it remains unclear what advantage is conferred by the basal location of these divisions, as terminal differentiative divisions can also occur at the apical surface in the zebrafish CNS [33,39]. Perhaps the basal location predisposes the division to asymmetric differentiative fates, while apical mitoses that generate two neurons may favour the generation of daughters with symmetric neuronal cell fates.

In addition to Vsx1-expressing NAPs, a small population of spinal NAPs express Olig2. Olig2 is expressed in apical progenitors and motor neurons before 24 hpf and in OPCs around the same time as NAPs in mitosis express Olig2 (36 hpf). This suggests that Olig2-expressing OPCs are probably derived from NAPs while motor neurons are more likely to be derived from apical progenitors [35].

Compared with the spinal cord, the hindbrain in zebrafish embryos contains more diverse NAPs. We find hindbrain NAPs in three locations (SA, VMB, DLB) and note these differ dynamically during development. The surface NAPs (SA), for example, increase during development while deeper NAPs (VMB) decrease through time. A previous study of zebrafish hindbrain demonstrated that neurons located close to the ventricular surface are born later than the ones located deeper in the neuronal layer, and the former regulate fine locomotor movements while the latter regulate the fastest movements [40]. Thus, although we have not followed the final fate of neurons derived from the deep and superficial NAPs, we speculate that neurons derived from late-dividing NAPs close to ventricular surface (SA) and the deeper, earlier-dividing VMB NAPs may produce neurons that regulate fine and fastest locomotor movements, respectively.

NAPs in the zebrafish telencephalon appear very distinct from NAPs in spinal cord or hindbrain, and remain the least well defined from our study. Although a small population expresses Vsx1 and Tbr2, the expression profile of 90% of telencephalic NAPs remains to be determined. In contrast with more caudal regions, we found most embryonic telencephalic NAPs divide in subapical positions of the neuroepithelia rather than in more basal locations. Considerably more work will be required to understand NAPs in zebrafish telencephalon.

We have been cautious in naming the non-apical progenitor populations in the zebrafish, in line with a recent discussion of progenitor names in the mammalian cortex that concluded that we do not yet understand enough about these cells to give them definitive names [41]. Thus, we have just called all zebrafish progenitors that do not undergo mitosis at the apical surface NAPs. As we find zebrafish NAPs in several distinct locations it may well be the case that zebrafish NAPs will eventually be classified into different subtypes, but currently we do not have sufficient understanding to do this.

Our observations and those of others [27] suggest that the majority of zebrafish spinal NAPs divide to produce two neurons. This neurogenic potential was further supported by the finding that zebrafish NAPs express the pan-neuronal marker HuC/D. In addition, when we promoted neurogenesis by overexpressing the DN-Su(H) construct, we observed that both NAP and neuronal populations were increased at the expense of apical neural progenitors. This suggests that zebrafish spinal NAPs are regulated in a very similar manner to neurons, and in this respect resemble mammalian basal progenitors [2,18]. We have so far been unable to find any evidence for bRGC in the zebrafish embryo. bRGC are to date the only NAPs capable of self-renewing and generating long-term cell lineages in mammalian cortex [7,9]. However, as all NAPs are rare in the zebrafish embryo CNS, we cannot rule out the existence of self-renewing NAPs in zebrafish CNS. Capturing such cells by random mosaic labelling and/or live imaging will be technically very challenging. There is still much to learn about basal progenitors in the embryonic teleost CNS.
